# Comparative Effectiveness of Synthetic and Natural Antioxidants on the Oxidative Stability of Lard

**DOI:** 10.3390/foods15173145

**Published:** 2026-09-04

**Authors:** Jixiao Jian, Huihui Zhang, Fengqin Tu, Xinghe Zhang, Jiaojiao Yin, Pan Gao

**Affiliations:** 1 Key Laboratory of Edible Oil Quality and Safety, State Administration for Market Regulation, Key Laboratory for Deep Processing of Major Grain and Oil of Ministry of Education in China, College of Food Science and Engineering, Wuhan Polytechnic University, Wuhan 430023, China; 2 Wuhan Institute for Food and Cosmetic Control, Wuhan 430012, China

**Keywords:** lard, tert-butylhydroquinone, propyl gallate, oxidative stability

## Abstract

The oxidative stability of animal fat is crucial for maintaining nutritional quality and food safety. This study aimed to evaluate the effects of four phenolic antioxidants (i.e., tert-butylhydroquinone, TBHQ; propyl gallate, PG; butylated hydroxytoluene, BHT; vitamin E, VE) on the stability of lard. The inhibitory effects of different antioxidant concentrations were comprehensively assessed using chemical index analyses, free radical scavenging assays, and electron paramagnetic resonance (EPR). The results showed that three synthetic antioxidants performed significantly better than VE, with TBHQ and PG exhibiting the best overall antioxidant performance. Specifically, TBHQ (0.2 g/kg) most effectively suppressed the increase in the peroxide value and was ranked first in the principal component analysis. PG significantly reduced the anisidine value (4.01) and demonstrated the best DPPH scavenging capacity (55.59 μmol TE/100 g). Notably, TBHQ presents certain toxicological concerns, whereas PG exhibits comparatively lower toxicity. Consequently, for practical industrial applications, PG should be prioritized as the antioxidant of choice for lard. Based on the EPR radical-scavenging results and known antioxidant chemistry, it was inferred that the antioxidants quenched superoxide anion radicals via hydrogen-atom and single-electron transfer mechanisms, thereby interrupting the oxidative chain reaction. This study provides a theoretical basis for the scientific selection of antioxidants in lard and offers guidance for ensuring food safety and nutritional quality.

## 1. Introduction

Lard is a traditional cooking fat that is widely used in Chinese cuisine and is primarily rendered from pork fat such as leaf and back [1]. It is suitable for most Chinese cooking methods, including stir- and deep-frying. Lard is highly digestible, easily absorbed, and contributes unique flavor and texture to dishes [2,3,4]. Owing to its high saturated fatty acid content [5], lard is semi-solid at room temperature and shows good plasticity and extensibility, making it particularly effective for generating flaky textures in traditional Chinese pastries [6]. Although lard exhibits a relatively high oxidative stability at room temperature, it has limited antioxidant capacity during storage and processing [7]. Oxidation significantly compromises the sensory quality, nutritional value, and safety of lard [8]. Furthermore, long-term excessive consumption of oxidized lard may be associated with increased blood cholesterol levels and a higher risk of cardiovascular disease [9].

To meet increasing consumer interest in high nutritional retention and minimal formation of hazardous substances in food, it is insufficient to rely solely on the inherent oxidative stability of lards. Fully sealed packaging of lard is challenging because of its semi-solid physical state, which results in prolonged exposure to oxygen and light during storage and use. Consequently, these factors lead to stricter requirements for the oxidative stability and storability of lards. Adding exogenous antioxidants to lard is a common and effective approach to address these challenges, in addition to conventional methods, such as avoiding light and oxygen exposure and temperature control [10]. Common antioxidants used in the oil industry include synthetic compounds, such as tert-butylhydroquinone (TBHQ), propyl gallate (PG), and butylated hydroxytoluene (BHT), as well as natural antioxidants, such as vitamin E (VE), each with distinct mechanisms of action and cost-effectiveness. Notably, the efficacy of the same antioxidant can vary among oils, which is likely attributable to differences in fatty acid profiles, trace chemical compositions, and possible residues from processing. For instance, studies have shown that TBHQ effectively scavenges peroxyl radicals through its active hydroxyl groups in rapeseed oil, thereby significantly inhibiting hydroperoxide formation [11]. In contrast, in olive oil rich in endogenous antioxidants such as tocopherols and polyphenols, TBHQ exhibited limited antioxidant efficacy owing to competitive reactions with these endogenous antioxidants [12]. Currently, research on the antioxidants in lard remains underexplored. Most existing studies have focused only on single indicators or concentrations and lack systematic comparisons among multiple antioxidants at various concentrations. Furthermore, the intrinsic mechanisms by which antioxidants influence free radical generation and interact with the inherent components of lard to delay oxidation have not been thoroughly investigated and warrant further elucidation using modern analytical techniques. Identifying effective concentration ranges and understanding the mechanisms of action of different antioxidants in lard under different storage conditions are important for guiding formulation design, optimizing storage strategies, and preserving the original quality and nutritional value of lard.

To address these research gaps, this study comparatively evaluated the effects of different antioxidants, PG (0.1 g/kg), TBHQ (0.1 and 0.2 g/kg), BHT (0.1 and 0.2 g/kg), and VE (0.1 and 0.2 g/kg), on the oxidative stability of lards. The Schaal oven tests, Rancimat analyses, free radical scavenging assays, and nutritional component analyses were conducted to compare the effects of these antioxidants. Electron paramagnetic resonance (EPR) was employed to evaluate the inhibitory effects of antioxidants on the oxidative progression of lard and to elucidate their antioxidant mechanisms. The findings revealed the efficacy differences and potential modes of action of these antioxidants in lards during storage, which will provide scientific evidence and theoretical insights for the selection of antioxidants and shelf-life prediction of lard products.

## 2. Materials and Methods

### 2.1. Materials and Chemicals

Lard was purchased from Taian Haizhirun Food Co., Ltd. (Shandong, Taian, China). PG was obtained from Henan Ruyi Biotechnology Co., Ltd. (Henan, Zhengzhou, China). TBHQ, BHT, and VE were supplied by Shanghai Yuanye Bio-Technology Co., Ltd. (Shanghai, China). The standards of 37 fatty acid methyl esters (FAMEs), α-, β-, γ-, and δ-tocopherols (purity > 98%), 6-hydroxy-2,5,7,8-tetramethylchromane-2-carboxylic acid (Trolox), 1,1-diphenyl-2-picrylhydrazyl (DPPH), ferric reducing antioxidant power (FRAP), 2,2′-azinobis(3-ethylbenzothiazoline-6-sulfonate) (ABTS), and 2,4,6-tris(2-pyridyl)-s-triazine (TPTZ) were purchased from Sigma-Aldrich Co., Ltd. (St. Louis, MO, USA). 5,5-Dimethyl-1-pyrroline N-oxide (DMPO, purity > 97%) and KBr (chromatographic grade, 99.5%) were purchased from Shanghai Aladdin Biochemical Technology Co., Ltd. (Shanghai, China). Other chemicals were provided by Sinopharm Chemical Reagent Co., Ltd. (Hubei, Wuhan, China).

### 2.2. Sample Preparation

According to the Codex Alimentarius Standards (CXS 192-1995, 2019 [13]), PG, TBHQ, and BHT were selected for comparative analysis at their maximum permitted levels in oil. The maximum permitted level of PG in edible fats and oils is 0.1 g/kg, while that of TBHQ and BHT is 0.2 g/kg. Specific concentrations used were PG (0.1 g/kg), TBHQ (0.1 g/kg and 0.2 g/kg), BHT (0.1 g/kg and 0.2 g/kg), and VE (0.1 g/kg and 0.2 g/kg), to evaluate their dose-dependent effects. Therefore, the antioxidant concentrations set in this study reflect practical regulatory-use scenarios, rather than a comparison of antioxidant potency at equimolar concentrations or across full dose–response gradients. Each antioxidant was accurately weighed and incorporated into the lard, followed by 15 min of ultrasonic-assisted dissolution to ensure a homogeneous dispersion. A control group without antioxidants was established in parallel.

### 2.3. Schaal Oven Test

Lard samples (200 g) were placed in beakers and then put in a constant-temperature oven (DHG-9245A, Yiheng, Shanghai, China) at 60 ± 1 °C to accelerate oxidation. The beakers were shaken every 12 h, and their positions were changed. Every 3 days, an appropriate amount of oxidized oil was taken for evaluation and analysis. All the processed samples were stored at 4 °C for analysis.

### 2.4. Fatty Acid Composition

The fatty acids in the lard were identified using gas chromatography (GC). Derivatization followed the protocol described by Santos et al. [14] with slight modifications. First, 0.1 g of the lard sample was mixed with 1 mL of an NaOH-methanol solution (0.5 mol/L) and shaken for 30 min. Then, 4 mL of n-hexane was added for extraction, followed by ultrasonic treatment for 10 min. The supernatant was collected for further analysis. The samples were analyzed using an Agilent 7890A gas chromatograph (Shanghai, China). Separation was performed on an HP-88 polycyanopropyl siloxane capillary column (100 m × 0.25 mm, 0.5 μm, Agilent), with an injection volume of 1 μL and a split ratio of 20:1. The temperature was set to 100 °C, then increased to 180 °C at a rate of 10 °C/min, held at 180 °C for 6 min, subsequently raised to 200 °C at 1 °C/min, and held for 20 min. Qualitative analysis was performed based on the retention times of the chromatographic peaks, while the content of each fatty acid component was quantified using the peak area normalization method.

### 2.5. Physicochemical Properties

Acid value (*AV*), peroxide value (POV), and p-anisidine value (p-AnV) were analyzed using the AOCS methods Cd 3d-63, Cd 8b-90, and Cd 18-90, respectively.

Determination of AV. First, 1.0 g of oil sample was dissolved in 50 mL of a diethyl ether-isopropanol solution and shaken for 2 min. Subsequently, the mixture was titrated with 0.01 mol/L NaOH using phenolphthalein as an indicator. The acid value of the lard sample was calculated using the formula below, where *v* represents the volume of NaOH solution consumed by the sample (mL), *c* is the concentration of the NaOH solution (mol/L), and *m* is the mass of the lard sample (g).

(1) $$AV(mg/g)=\frac{56.1vc}{m}$$

Determination of POV. An accurately weighed 2.0 g of lard sample was dissolved in 50 mL of a glacial acetic acid–isooctane mixture (3:2, *v*/*v*), followed by the addition of 0.5 mL of saturated KI solution. After thorough mixing, the mixture was shaken in the dark for 1 min and diluted with 50 mL of distilled water. The resulting mixture was immediately titrated vigorously with 0.01 mol/L Na_2_S_2_O_3_ standard solution until a pale-yellow color appeared. Subsequently, 0.5 mL of sodium dodecyl sulfate solution (10%) and 0.5 mL of starch indicator (10%) were added, and titration was continued until the blue color disappeared. Blank titrations were performed using reagents as controls. KI, Na_2_S_2_O_3_, and starch solutions were all freshly prepared before testing. Peroxide concentration (meq/kg) was calculated using the equation below, where *c* represents the concentration of Na_2_S_2_O_3_ (mol/L), *v* is the volume of Na_2_S_2_O_3_ consumed by the sample (mL), *v*_0_ is the volume of Na_2_S_2_O_3_ consumed by the blank (mL), and *m* is the mass of the lard sample (g).

(2) $$POV(meq/kg)=\frac{(v-v_{0})c}{m}\times 1000$$

Determination of p-AnV. An accurately weighed 1.5 g of oil sample was placed in a 25-mL volumetric flask, dissolved in isooctane, and diluted to the mark. The solution was then thoroughly mixed. The absorbance *A*_1_ of the isooctane solution was measured at 350 nm using a 1 cm cuvette. An aliquot of 5 mL of the isooctane solution was transferred into a 10 mL volumetric flask, and 1 mL of p-anisidine reagent was added (0.25% *w/v* in glacial acetic acid, freshly prepared). After mixing, the solution was allowed to react in the dark for 10 min. The absorbance *A*_2_ was then measured at 350 nm using a 1 cm cuvette. For the blank, 5 mL of isooctane was used instead of the sample solution, mixed with 1 mL of p-anisidine reagent, and the absorbance *A*_3_ was measured after the same 10 min reaction. The p-anisidine content was calculated using the formula below, where 25 is the volume of isooctane used for the dilution (mL), 1.2 is the dilution correction factor, and m is the sample mass (g).

(3) $$p-AnV=\frac{25\times 1.2\times (A_{2}-A_{1}-A_{3})}{m}$$

### 2.6. Antioxidant Capacity

The antioxidant activity of lard was determined using a modified method from Gao et al. [15]. A lard sample at 1.5 g was accurately weighed and homogenized with 3 mL of methanol. The mixture was vortexed for 20 min under light-protected conditions and centrifuged at 3750 r/min for 3 min. The resulting supernatant was collected in a brown glass vial as the polar fraction of lard, whereas the remaining residue represented the nonpolar fraction. For the whole-oil fraction, 0.15 g of lard was dissolved in 3 mL of ethyl acetate to prepare a sample solution with a concentration of 15 mg/mL. Antioxidant activity was determined using three distinct assays: ABTS, FRAP, and DPPH radical scavenging. The results were quantified using a Trolox standard curve (μmol TE/100 g).

#### 2.6.1. DPPH

A test sample solution of 2 mL was mixed with 2 mL of the DPPH–methanol working solution (0.1 mmol/L). The mixture was vortexed and allowed to react in the dark for 2 h. Subsequently, the absorbance was measured at 517 nm using a UV-visible spectrophotometer (UV755B, Shanghai Yidian Analytical Instrument Co., Ltd., Shanghai, China). To analyze the DPPH radical scavenging activity of the non-polar components or whole oil, ethyl acetate was used as the solvent system instead of methanol. All results were expressed as Trolox equivalents (μmol TE/100 g). The radical scavenging rate (*R*) was calculated using the formula below: *A*_1_ was the absorbance of 2.0 mL of blank solvent (methanol for polar fraction, or ethyl acetate for non-polar and whole-oil fractions) mixed with 2.0 mL of DPPH standard solution; *A*_2_ was the absorbance of 2.0 mL of DPPH standard solution mixed with 2.0 mL of oil sample extract; and *A*_3_ was the absorbance of 2.0 mL of blank solvent (methanol for polar fraction, or ethyl acetate for non-polar and whole-oil fractions) mixed with 2.0 mL of oil sample extract. In parallel, a control experiment was performed by replacing the 2.0 mL oil sample extract in the reaction mixture with a 2.0 mL Trolox standard solution.

(4) $$R(\%)=\left(1-\frac{A_{2}-A_{3}}{A_{1}}\right)\times 100\%$$

#### 2.6.2. ABTS

The ABTS radical stock solution was prepared by mixing ABTS solution with potassium persulfate solution at a volume ratio of 625:11. The mixture was then incubated in the dark at room temperature for 12 h. This stock solution was diluted with methanol until an absorbance of 0.70 ± 0.02 at 734 nm was achieved to obtain the ABTS working solution. In the assay, 2 mL of this working solution was mixed with 100 μL of polar extract in a brown sample vial. After reacting in the dark at room temperature for 20 min, the absorbance was measured at 734 nm. For the construction of the standard curve, 100 μL of polar extract was replaced with an equal volume of a series of Trolox standard solutions, which were assayed simultaneously. The results were expressed as micromoles of Trolox equivalents per 100 g of sample (μmol TE/100 g). The radical scavenging rate (*R*) was calculated using the formula below: the sample group *A*_1_ contained ABTS working solution mixed with polar extract, while the control group *A*_2_ consisted solely of ABTS working solution for baseline measurement.

(5) $$R(\%)=\left(\frac{A_{2}-A_{1}}{A_{2}}\right)\times 100\%$$

#### 2.6.3. FRAP

The FRAP working solution was freshly prepared by mixing 20 mmol/L FeCl3 solution, 10 mmol/L TPTZ solution, and 0.1 mol/L sodium acetate buffer (pH 3.6) in a volume ratio of 1:1:10. Then, 2 mL of the FRAP working solution was thoroughly mixed with 300 μL of the polar extract. The mixture was allowed to react at room temperature in the dark for 10 min, after which absorbance was measured at 593 nm. To construct a standard curve, the polar extract was replaced with an equal volume of the Trolox (a water-soluble vitamin E analog) standard solution, which was assayed simultaneously. The results were expressed as Trolox equivalents (μmol TE/100 g).

#### 2.6.4. Rancimat Test

The Rancimat test was performed using an 892 oxidative stability instrument (Metrohm, Switzerland) at 120 °C, and the flow rate of air was 20 L/h. A lard sample size of 3.00 ± 0.01 g was utilized. Oxidative stability was expressed as an oxidative stability index (OSI) and the antioxidant protection factor was calculated. The calculation formula is as follows: the protection factor (*PI*) was calculated as the ratio of the oxidative induction time of an oil sample containing antioxidants (*IP_X_*) to that of an oil sample without antioxidants (*IP*_0_).

(6) $$PI=\frac{{IP}_{X}}{{IP}_{0}}$$

### 2.7. Tocopherol

An Agilent 1200 HPLC system (1260, Agilent, Santa Clara, USA) equipped with a Nucifera Si 60 silica column (250 mm × 4.6 mm, 5 μm; Hanbon, Henan, China) was used to determine the tocopherol content of the lard using the method described in ISO 9936 [16]. The oil sample of 1.0 g was accurately weighed and mixed with 0.1 g BHT and 10 mL of the mobile phase. The mixture was dissolved using ultrasonic oscillation and subsequently diluted to a certain volume using the mobile phase. The mobile phase consisted of n-hexane and isopropanol (98.5:1.5, *v*/*v*) with a flow rate of 1 mL/min. The excitation wavelength, emission wavelength, injection volume, and detection temperature were 294 nm, 328 nm, 10 μL, and 30 °C, respectively.

### 2.8. Free Radical Measurement

Free radical measurements were performed according to the method described by Huang et al. [17], with slight modifications. A portion of the sample was mixed with DMPO and drawn into a ring-marked capillary tube to a height greater than 20 mm. After sealing the capillary on a wax sealing plate, it was placed in an NMR tube containing a solution of pH3.6. The sample was subsequently positioned in the resonant cavity of an electron paramagnetic resonance spectrometer (EPR, 5000, Bruker, Ettlingen, Germany) for measurement. The instrumental parameters were set as follows: center field, 335 mT; sweep width, 20 mT; sweep time, 60 s; modulation amplitude, 0.22 mT; modulation frequency, 100 kHz; and five accumulated scans.

### 2.9. Statistical Analysis

Data analyses were conducted using Origin 2021 (OriginLab Corporation, Northampton, MA, USA) and SPSS 27.0 (IBM SPSS Statistics, Beijing, China). All independent experiments were performed in triplicate (n = 3), with results presented as mean ± standard deviation. Statistical comparisons were carried out using one-way analysis of variance (ANOVA) with Duncan’s post hoc test at a 5% significance level. Statistical significance was set at *p* < 0.05.

## 3. Results and Discussion

### 3.1. Fatty Acid Compositions

Table 1 presents the fatty acid composition of lard at 0, 6, and 15 days. The fatty acids in lard were primarily composed of monounsaturated fatty acids (MUFAs, 45.96–46.72%) and saturated fatty acids (SFAs, 35.59–36.94%), with polyunsaturated fatty acids accounting for only 14.23–14.75%. These findings were consistent with those reported by Pop and Dippong [18]. Oleic acid (C18:1, 43.02–44.88%) was the main constituent of monounsaturated fatty acids. The PUFAs were predominantly linoleic acid (C18:2, 13.19–13.73%), with a small amount of linolenic acid (C18:3, 1.02–1.18%), whereas the SFAs were mainly palmitic and stearic acids (C16:0, 23.14–23.98%; C18:0, 11.10–11.93%), along with minor amounts of myristic acid (C14:0, 1.24–1.29%). The addition of various antioxidants did not significantly alter the fatty acid profile of lard, indicating that they did not change the type or fundamental composition of the fatty acids. Throughout the accelerated oxidation process conducted in an oven, the fatty acid composition of the lard remained relatively stable, with the proportion of SFAs consistently lower than that of unsaturated fatty acids. Although dominant MUFAs serve as primary targets for oxidation, the reactivity of their single double bonds is relatively limited. PUFAs, characterized by their high reactivity, were initially present at low concentrations in the lard. This specific compositional profile results in an insufficient total concentration of highly reactive double-bond sites within the system, thereby limiting both the extent and rate of the oxidation reaction.

### 3.2. Physicochemical Indices

#### 3.2.1. AV

**Figure 1:**
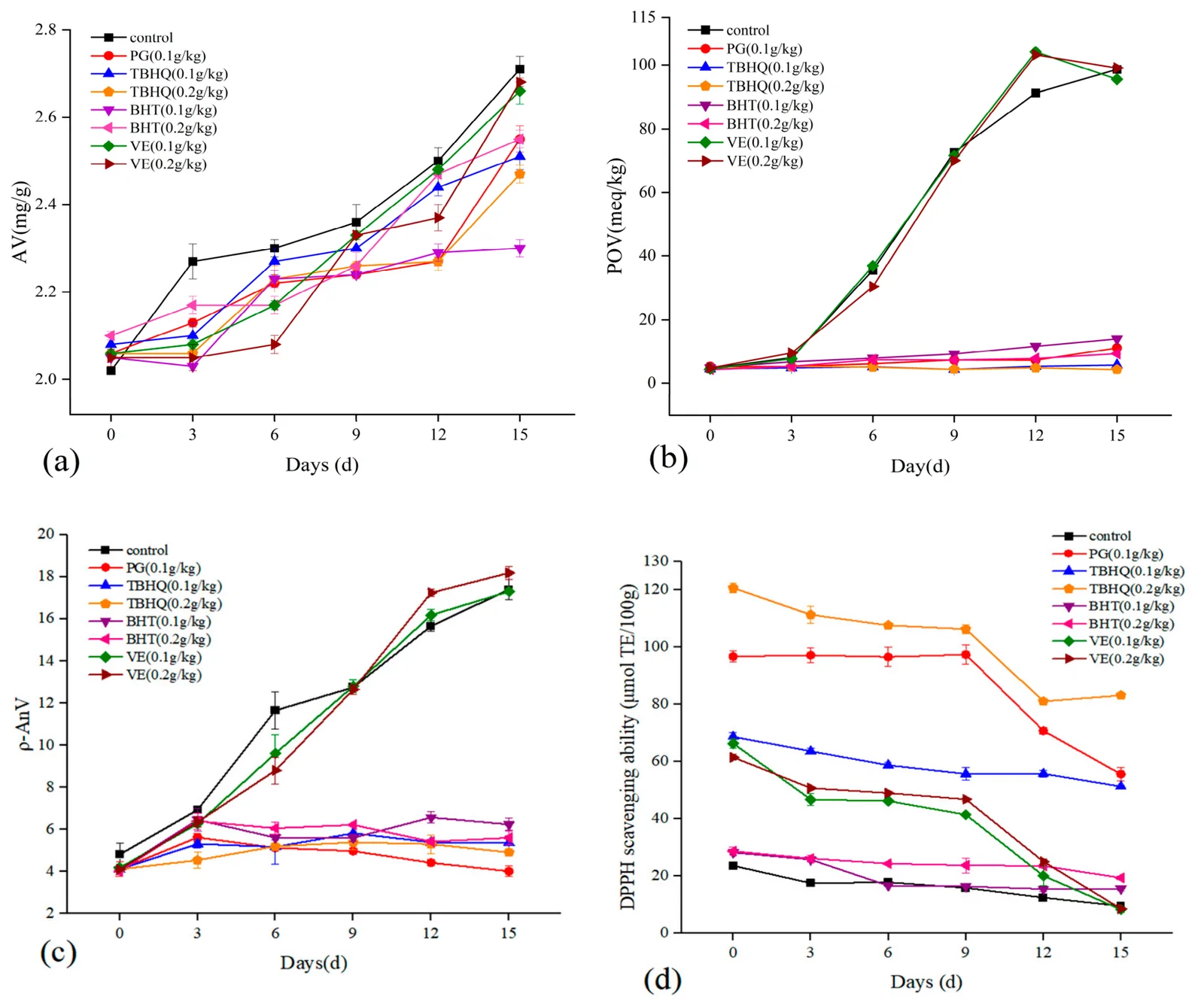
Effect of different antioxidants (g/kg) on the AV of lard (**a**); effect of different antioxidants (g/kg) on the POV of lard (**b**); effect of different antioxidants (g/kg) on the p-AnV of lard (**c**); effect of different antioxidants (g/kg) on the DPPH scavenging ability of lard (**d**).

As shown in Figure 1a, the AV of lard demonstrated a significant upward trend during accelerated oxidation at 60 °C. In the absence of antioxidants, the AV of lard increased markedly more rapidly during the initial 0–3 days, reaching 2.71 mg KOH/g after 15 days and surpassing the threshold established by the Codex Alimentarius Commission global standard (≤1.5 mg KOH/g). Samples containing antioxidants showed significantly lower AV than the control group, indicating that the antioxidants effectively suppressed the increase in AV during oxidation. However, VE exhibited limited efficacy in inhibiting the increase in AV under high-temperature conditions. At the highest dose tested (0.2 g/kg), VE exhibited inhibitory effects on AV only within the first 9 days; beyond this period, its effectiveness was significantly diminished. This limited efficacy can be attributed to VE’s role of vitamin E as a chain-breaking antioxidant. Under accelerated oxidation conditions at 60 °C, the compound consistently engaged in antioxidant reactions, leading to its gradual depletion over time. Consequently, its concentration gradually decreases, rendering it unable to suppress the continued increase in AV resulting from hydrolytic and oxidative rancidity. Based on the final AV measured after 15 days, BHT showed the lowest value (2.30 mg KOH/g). The increase in AV primarily originates from the hydrolysis of triglycerides, facilitated by moisture, thermal energy, or acidic or basic conditions, leading to ester bond cleavage and the release of free fatty acids [19]. Although oxidation processes can produce acidic byproducts such as low-molecular-weight carboxylic acids, their impact on the overall AV is generally minimal. Under the controlled conditions of the Schaal oven test, which primarily accelerated the formation of hydroperoxides, the accumulation of secondary oxidation products remained relatively low. Consequently, acidic substances generated through oxidative pathways did not significantly affect the AV [20].

#### 3.2.2. POV

To evaluate the formation of primary oxidation products during accelerated storage, the POV of the lard was measured, and the results are shown in Figure 1b. In the absence of antioxidants, the POV of lard exhibited a pronounced increasing trend over the 15-day accelerated oxidation period, peaking at 99.05 meq/kg. This indicated that significant primary oxidation occurred in the lard under 60 °C conditions, resulting in substantial accumulation of hydroperoxides. Conversely, when PG, BHT, and TBHQ were introduced, the POV of lard remained at a lower and more stable level, ranging from 4.49 meq/kg to 13.95 meq/kg, which was only 14.08% of the POV observed in the control group. This indicates that all three antioxidants exerted a significant inhibitory effect on the increase in POV in lard during storage. This result is consistent with previous findings that phenolic antioxidants effectively inhibit the formation of primary oxidation products in olive oil [21]. In contrast, the addition of VE did not significantly affect the POV of the lard, indicating limited antioxidant efficacy. This phenomenon may be attributed to the generation of oxidative radicals by VE under 60 °C conditions, potentially leading to pro-oxidant activity [22].

#### 3.2.3. p-AnV

Generally, p-AnV reflects the concentration of secondary oxidation products such as aldehydes and ketones [23]. As shown in Figure 1c, the p-AnV of lards without antioxidants increased progressively with storage time. The addition of VE did not significantly inhibit the increase in p-AnV; at 15 days, both the control (17.39) and VE-treated groups (0.1 g/kg: 17.30; 0.2 g/kg: 18.18) exhibited relatively high values. In contrast, when PG, TBHQ, or BHT were added, the p-AnV of lard remained stable during oxidation, ranging from 4.01 to 6.24, representing only 23.06% to 35.88% of the control group’s p-AnV. Among these antioxidants, PG resulted in the lowest p-AnV, demonstrating that PG effectively suppressed the formation of secondary oxidation products in lard.

### 3.3. Antioxidant Capacity

The DPPH radical assay is extensively employed to assess the free radical scavenging activity of antioxidants, as it forms a stable molecule upon accepting an electron or a hydrogen atom from the antioxidants [24]. As shown in Figure 1d, the trend of the DPPH radical scavenging capacity in lard suggests that the addition of antioxidants significantly enhanced the free radical scavenging ability. The addition of PG and TBHQ resulted in the most pronounced effects. At equal concentrations (0.1 g/kg), PG exhibited the strongest antioxidant capacity (96.74 μmol TE/100 g) and maintained the highest free radical scavenging activity after 15 days of oxidation (55.59 μmol TE/100 g). However, TBHQ (0.2 g/kg) outperformed PG at the highest concentration tested, indicating a clear dose-dependent relationship with the antioxidant efficacy. In contrast, although VE significantly enhanced the antioxidant capacity of lard in the short term (up to 12 days, 61.48–66.36 μmol TE/100 g), its stability was compromised by accelerated high-temperature oxidation. VE was susceptible to autoxidative degradation and might even exhibit pro-oxidative effects under certain conditions, resulting in no significant difference in free radical scavenging ability compared to the control group by the end of the oxidation period. BHT showed the weakest improvement in antioxidant capacity (28.19–28.65 μmol TE/100 g), which remained at a similarly low level as that of the control group throughout the oxidation process.

Table 2 presents the evaluation results of the antioxidant capacity when different antioxidants were added to lard. At an addition level of 0.1 g/kg, assessments by the DPPH, ABTS, and FRAP methods indicated that the free radical scavenging capacities of the antioxidants in lard followed the order: PG > TBHQ > BHT > VE. This ranking was primarily attributed to the differences in their molecular structures. PG, a phenolic antioxidant containing three hydroxyl groups, can release a large number of active hydrogen ions from the hydroxyl groups on its benzene ring, which combine with DPPH radicals possessing a lone pair of electrons, thereby exhibiting a higher free radical scavenging rate [25]. At an addition level of 0.2 g/kg, TBHQ exhibited the strongest free radical scavenging capacity across all assay systems, indicating that the antioxidant efficacy of TBHQ is markedly concentration-dependent. Therefore, the superior performance of TBHQ at 0.2 g/kg should be attributed to the synergistic effect of its intrinsic activity and the elevated dosage, rather than to an inherently greater potency of TBHQ compared with PG. However, in the polar DPPH system, PG exhibited a higher free radical scavenging ability than TBHQ, likely because of the reactivity of its polyphenolic structure in polar environments.

Using the ABTS method, which evaluates the antioxidant capacity through a single-electron transfer mechanism, BHT showed the second-highest free radical scavenging capacity after TBHQ at 0.2 g/kg. This was because BHT contains three electron-donating groups (two tert-butyl groups and one methyl group). These groups can enhance the stability of phenoxyl radicals through the delocalization of unshared electron pairs in the p-orbitals and the electron-donating effect, while increasing the electron cloud density of the benzene ring and reducing the dissociation energy of the phenolic hydroxyl group, thereby improving its free radical scavenging capacity [26]. In lard, PG exhibited an OSI of 6.27 h and a PI of 7.74, indicating strong antioxidant efficacy, consistent with the results of the FRAP, ABTS, and DPPH assays.

### 3.4. Tocopherol Content

Tocopherol served important nutritional roles such as preventing low-density lipoprotein oxidation and protecting lipids from rancidity during storage [27]. As shown in Table 3, tocopherols were detected only in the VE-supplemented lard samples, with total contents of 90.41 mg/kg and 176.19 mg/kg, respectively. After accelerated oxidation at 60 °C, only α-tocopherol remained detectable in the system. The rapid depletion of tocopherols may be attributed to their continuous consumption during the antioxidant process, in which α-tocopherol delays lipid oxidation by interrupting oxidative chain reactions and quenching reactive oxygen species. As the α-tocopherol concentration decreases, its oxidation rate increases, thereby accelerating its degradation [28]. However, this explanation remains speculative and was not directly verified in the present study. The antioxidant activity of VE is influenced by factors such as the surrounding medium, environmental conditions, and concentration. The primary mechanism involves capturing or neutralizing free radicals to inhibit oxidation, thereby preserving food quality and nutritional components [29]. Adding VE to oil boosts the tocopherol content and protects the oil and its nutrients from lipid oxidation. However, their antioxidant effects are mainly against free radicals and offer limited protection against oxidative damage caused by non-radical species to other nutrients. Therefore, an appropriate addition level is essential for maximizing the antioxidant efficacy of VE, safeguarding the quality and stability of the oil, and enhancing its nutritional value.

### 3.5. Free Radical Content

**Figure 2:**
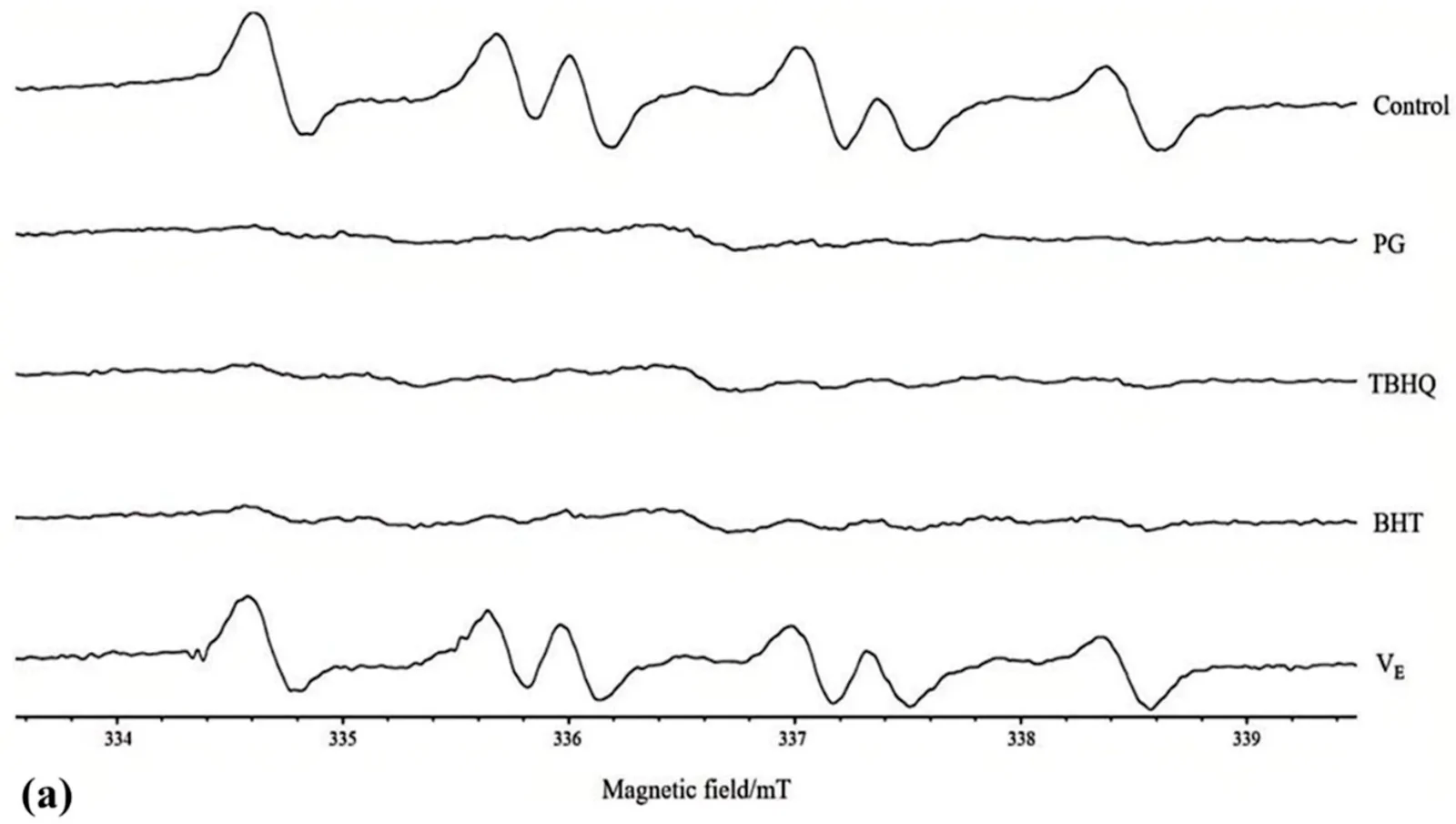

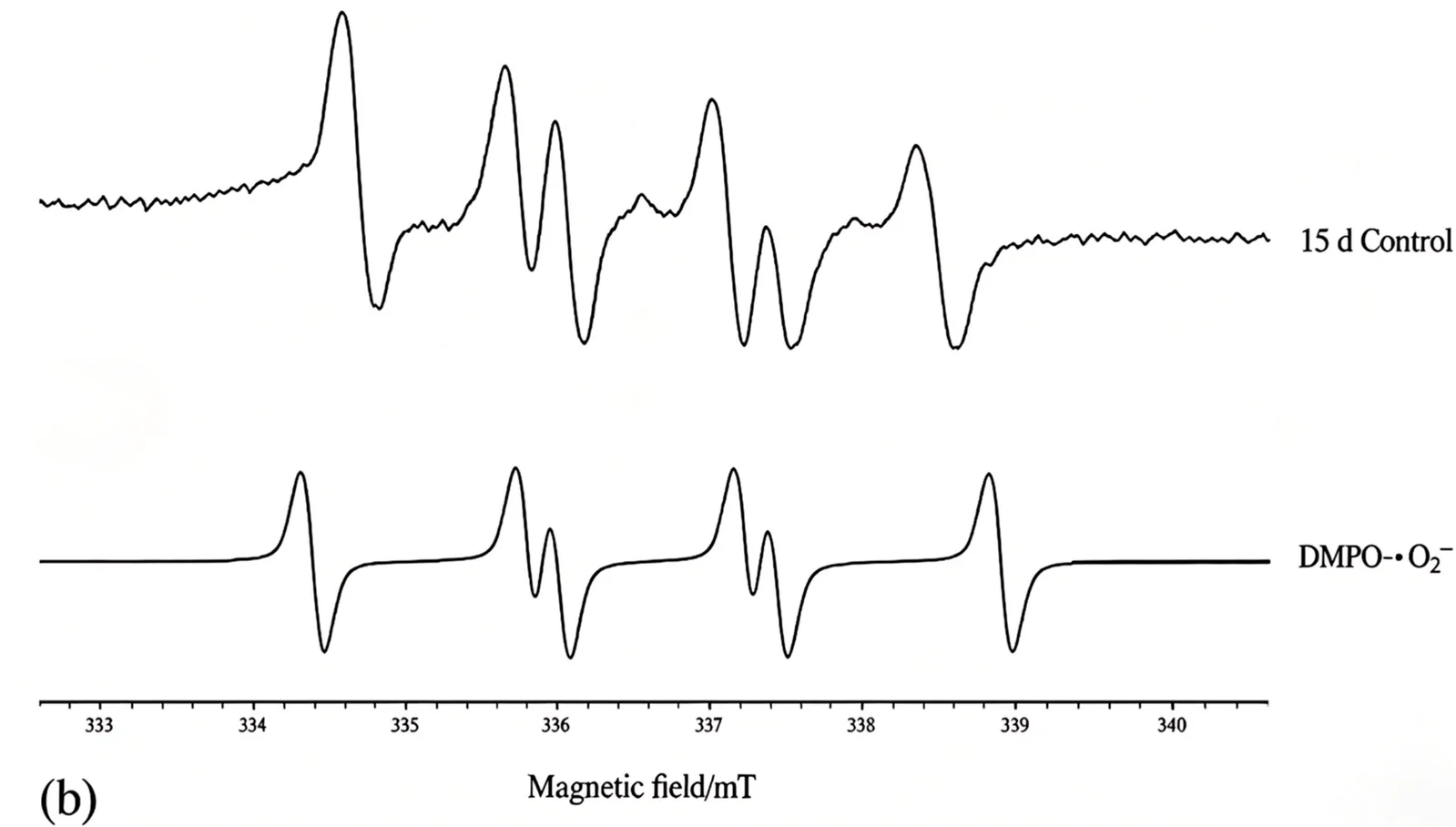
Effects of different antioxidants on changes in free radicals after accelerated oxidation of lard (**a**); lard fitting map after accelerated oxidation (**b**).

Figure 2a,b show the fitted EPR spectra of lard and free radical spectra of lard with antioxidants after accelerated oxidation. A comparison between the experimental and fitted spectra revealed that the primary radical species generated in lard after accelerated oxidation was the superoxide anion radical (•O_2_^−^). The superoxide anion radical is a key reactive species produced in oxidative cascade reactions and can be converted into stronger prooxidants [30]. It can activate transition metals through redox cycles (e.g., Fe^3+^/Fe^2+^) and undergo dismutation to generate H_2_O_2_, which subsequently leads to the formation of highly oxidizing hydroxyl radicals (•OH) via Fenton-like reactions. Hydroxyl radicals can abstract allylic hydrogen atoms from polyunsaturated fatty acids, generating lipid radicals (L•), and initiating autoxidation chain reactions [31]. Based on the free radical absorption intensity, it is evident that the addition of antioxidants effectively suppressed radical formation. Antioxidants significantly reduced free radical absorption in the lard group compared to the control group. VE exhibited the weakest inhibitory effect, whereas the three synthetic antioxidants showed stronger radical suppression. Therefore, they could only partially inhibit free radical generation rather than completely prevent it.

### 3.6. Principal Component Analysis (PCA)

Principal component analysis (PCA) was employed to evaluate the relationships among quantitative indicators, including AV, POV, p-AnV, DPPH radical-scavenging capacity, FRAP, ABTS, and DPPH (polar and non-polar) radical-scavenging capacities, of lard samples supplemented with different antioxidants after 15 days of accelerated oxidation. The first two principal components had eigenvalues of λ_1_ = 7.828 and λ_2_ = 1.275, accounting for 78.28% (PC1) and 12.75% (PC2) of the total variance, respectively, with a cumulative contribution rate of 91.03%. These results indicate that these principal components adequately represent the overall variability of the oil samples.

Table 4 presents the PCA score rankings of various antioxidants in the lard system. Comprehensive scores ranked TBHQ first, followed by PG and VE. This ranking was highly consistent with the single-indicator evaluation results of the physicochemical indices and antioxidant capacities in this study, further confirming that TBHQ and PG are suitable antioxidants for the lard system under the current experimental conditions. Both effectively inhibited the increase in AV, POV, and p-AnV, protected endogenous nutrients, and significantly enhanced the oxidative stability of lard. Notably, although TBHQ achieved a slightly higher comprehensive score than PG, PG performed comparably to TBHQ and outperformed it in certain core antioxidant indicators. For example, PG scored higher than TBHQ in DPPH scavenging capacity, FRAP reducing power, and OSI assays. This demonstrated its efficient and broad-spectrum radical scavenging ability across different reaction mechanisms. From a molecular structure perspective, PG contains three phenolic hydroxyl groups, whereas TBHQ contains only two. This structural difference allows PG to donate more active hydrogen atoms, increasing its radical scavenging capacity per molecule, and enabling faster interruption of the chain reaction during the initial burst phase of lipid oxidation. Furthermore, PG achieves desirable antioxidant effects at a low concentration (0.1 g/kg), aligning with the ‘as low as a reasonably practicable’ safety principle in food additive usage. Although TBHQ exhibited strong antioxidant efficacy in the present study, its application is subject to certain regulatory restrictions, and safety concerns have been documented in the toxicological literature. PG demonstrated antioxidant capacity comparable to TBHQ and outperformed it in several key evaluation indicators, while exhibiting relatively lower toxicity and a more favorable safety profile [32]. In conclusion, TBHQ and PG showed excellent antioxidant performances in the lard system, with minimal score differences; however, PG provided higher safety and broader regulatory acceptance with comparable efficacy. Consequently, from the perspectives of toxicological safety and long-term application, PG has emerged as a viable and safer alternative to TBHQ for antioxidant protection in lards.

## 4. Conclusions

This study systematically evaluated the effects of four phenolic antioxidants (TBHQ, PG, BHT, and VE) on the oxidative stability of lard under accelerated oxidation conditions at 60 °C. These findings indicated that antioxidants significantly enhanced the antioxidant capacity of lard by suppressing free radical generation and inhibiting oxidative chain reactions. TBHQ and PG demonstrated the best antioxidant performances by transferring phenolic hydroxyl hydrogen atoms and undergoing quinonoid transformation. Based on the EPR radical-scavenging results and known antioxidant chemistry, it was inferred that these antioxidants quench superoxide anion radicals via hydrogen-atom transfer and single-electron transfer mechanisms, thereby protecting unsaturated fatty acids and nutrients from oxidative damage and reducing harmful oxidation products. Owing to the known toxicity of TBHQ, PG was recommended as a safer alternative. Higher concentrations generally enhanced antioxidant efficacy, although each antioxidant behaved differently. Although VE showed moderate short-term free radical scavenging activity, it was unstable at high temperatures, leading to rapid self-oxidation and potential pro-oxidant effects. Consequently, its contribution to the enhancement of the long-term oxidative stability of lard was limited. It should be noted that this study employed an accelerated oxidation model at 60 °C, which, although widely used for rapid screening of antioxidants, may not fully reflect the actual oxidation behavior under long-term ambient storage conditions. While the improvement in oxidative stability may contribute to the preservation of quality attributes during storage, sensory and safety aspects were not directly evaluated in this study. Future research should validate these findings under ambient storage conditions, incorporate sensory evaluation, and explore the interactions with other bioactive components in lard, aiming to establish a synergistic nutrient protection system. This will provide stronger theoretical and practical support for advancing the scientific foundation of oxidative stability in food lipids.

## Figures and Tables

**Table 1:** Effects of different antioxidants (g/kg) on the fatty acid composition of lard.

Days		C14:0	C16:0	C16:1	C18:0	C18:1	C18:2	C18:3	SFA	MUFA	PUFA
0	Control	1.34 ± 0.05 ^a^	23.15 ± 0.04 ^ef^	2.16 ± 0.00 ^abc^	11.10 ± 0.02 ^h^	44.21 ± 0.09 ^fgh^	13.33 ± 0.01 ^cd^	1.18 ± 0.05 ^a^	35.59 ± 0.01 ^i^	46.37 ± 0.08 ^bcdef^	14.51 ± 0.06 ^b^
	PG/0.1	1.23 ± 0.01 ^c^	23.24 ± 0.09 ^def^	2.19 ± 0.00 ^ab^	11.61 ± 0.05 ^def^	44.10 ± 0.07 ^gh^	13.57 ± 0.01 ^ab^	1.04 ± 0.02 ^b^	36.08 ± 0.15 ^efgh^	46.29 ± 0.06 ^cdefgh^	14.61 ± 0.01 ^ab^
	TBHQ/0.1	1.23 ± 0.01 ^c^	23.28 ± 0.06 ^def^	2.16 ± 0.04 ^abc^	11.70 ± 0.05 ^cde^	44.07 ± 0.04 ^h^	13.60 ± 0.04 ^ab^	1.04 ± 0.02 ^b^	36.21 ± 0.02 ^cdefgh^	46.23 ± 0.00 ^cdefghi^	14.64 ± 0.02 ^ab^
	TBHQ/0.2	1.25 ± 0.03 ^bc^	23.14 ± 0.01 ^f^	2.16 ± 0.02 ^abc^	11.59 ± 0.03 ^ef^	44.38 ± 0.22 ^cdefg^	13.73 ± 0.15 ^a^	1.02 ± 0.00 ^b^	35.98 ± 0.06 ^gh^	46.54 ± 0.19 ^abcd^	14.75 ± 0.15 ^a^
	BHT/0.1	1.27 ± 0.02 ^bc^	23.33 ± 0.22 ^def^	2.13 ± 0.00 ^bc^	11.57 ± 0.04 ^efg^	44.40 ± 0.08 ^cdefg^	13.69 ± 0.04 ^a^	1.03 ± 0.00 ^b^	36.16 ± 0.27 ^defgh^	46.53 ± 0.07 ^abcde^	14.72 ± 0.04 ^ab^
	BHT/0.2	1.23 ± 0.01 ^c^	23.18 ± 0.00 ^def^	2.19 ± 0.00 ^ab^	11.59 ± 0.00 ^ef^	44.03 ± 0.03 ^h^	13.57 ± 0.00 ^ab^	1.03 ± 0.00 ^b^	36.00 ± 0.01 ^fgh^	46.22 ± 0.03 ^defghi^	14.60 ± 0.00 ^ab^
	V_E_/0.1	1.23 ± 0.01 ^c^	23.23 ± 0.05 ^def^	2.19 ± 0.01 ^ab^	11.57 ± 0.01 ^efg^	44.02 ± 0.01 ^h^	13.57 ± 0.00 ^ab^	1.02 ± 0.01 ^b^	36.03 ± 0.04 ^fgh^	46.21 ± 0.02 ^efghi^	14.59 ± 0.00 ^ab^
	V_E_/0.2	1.32 ± 0.08 ^ab^	23.20 ± 0.08 ^def^	2.18 ± 0.02 ^ab^	11.56 ± 0.01 ^efg^	44.06 ± 0.03 ^h^	13.44 ± 0.13 ^bc^	1.07 ± 0.06 ^b^	36.08 ± 0.00 ^efgh^	46.24 ± 0.01 ^cdefghi^	14.52 ± 0.07 ^b^
6	Control	1.22 ± 0.00 ^c^	23.37 ± 0.03 ^def^	2.23 ± 0.03 ^ab^	11.81 ± 0.07 ^abc^	44.14 ± 0.01 ^fgh^	13.19 ± 0.08 ^d^	1.04 ± 0.00 ^b^	36.40 ± 0.10 ^cde^	46.37 ± 0.02 ^bcdef^	14.24 ± 0.08 ^c^
	PG/0.1	1.22 ± 0.00 ^c^	23.19 ± 0.05 ^def^	2.25 ± 0.01 ^a^	11.49 ± 0.06 ^fg^	44.42 ± 0.31 ^cdef^	13.66 ± 0.08 ^ab^	1.04 ± 0.00 ^b^	35.90 ± 0.01 ^hi^	46.67 ± 0.30 ^ab^	14.69 ± 0.08 ^ab^
	TBHQ/0.1	1.23 ± 0.01 ^c^	23.35 ± 0.01 ^def^	2.07 ± 0.07 ^c^	11.51 ± 0.08 ^fg^	44.65 ± 0.09 ^abc^	13.66 ± 0.08 ^ab^	1.02 ± 0.01 ^b^	36.08 ± 0.19 ^efgh^	46.72 ± 0.15 ^a^	14.68 ± 0.09 ^ab^
	TBHQ/0.2	1.23 ± 0.01 ^c^	23.43 ± 0.03 ^cd^	1.69 ± 0.02 ^d^	11.81 ± 0.03 ^abc^	44.27 ± 0.01 ^efgh^	13.58 ± 0.00 ^ab^	1.02 ± 0.01 ^b^	36.47 ± 0.01 ^bcd^	45.96 ± 0.01 ^i^	14.60 ± 0.01 ^ab^
	BHT/0.1	1.23 ± 0.00 ^c^	23.37 ± 0.03 ^def^	1.65 ± 0.02 ^d^	11.76 ± 0.08 ^bcd^	44.30 ± 0.03 ^defgh^	13.67 ± 0.09 ^a^	1.02 ± 0.00 ^b^	36.35 ± 0.11 ^cdef^	45.96 ± 0.01 ^i^	14.70 ± 0.09 ^ab^
	BHT/0.2	1.24 ± 0.01 ^c^	23.29 ± 0.05 ^def^	1.63 ± 0.00 ^d^	11.49 ± 0.02 ^fg^	44.76 ± 0.07 ^ab^	13.67 ± 0.09 ^a^	1.02 ± 0.00 ^b^	36.02 ± 0.02 ^fgh^	46.39 ± 0.08 ^bcdef^	14.69 ± 0.09 ^ab^
	V_E_/0.1	1.24 ± 0.01 ^c^	23.66 ± 0.02 ^b^	1.61 ± 0.02 ^d^	11.90 ± 0.08 ^ab^	44.61 ± 0.08 ^abc^	13.29 ± 0.02 ^cd^	1.02 ± 0.00 ^b^	36.79 ± 0.09 ^ab^	46.22 ± 0.09 ^cdefghi^	14.31 ± 0.02 ^c^
	V_E_/0.2	1.23 ± 0.00 ^c^	23.64 ± 0.04 ^bc^	1.59 ± 0.00 ^d^	11.93 ± 0.05 ^a^	44.60 ± 0.06 ^abcd^	13.19 ± 0.08 ^d^	1.03 ± 0.01 ^b^	36.80 ± 0.09 ^ab^	46.19 ± 0.06 ^fghi^	14.23 ± 0.07 ^c^
15	Control	1.25 ± 0.01 ^bc^	23.22 ± 0.01 ^def^	1.68 ± 0.03 ^d^	11.83 ± 0.02 ^abc^	44.64 ± 0.04 ^abc^	13.23 ± 0.08 ^d^	1.02 ± 0.02 ^b^	36.29 ± 0.02 ^cdefg^	46.32 ± 0.01 ^cdefg^	14.24 ± 0.06 ^c^
	PG/0.1	1.23 ± 0.00 ^c^	23.21 ± 0.08 ^def^	1.68 ± 0.03 ^d^	11.59 ± 0.07 ^ef^	44.55 ± 0.05 ^bcde^	13.69 ± 0.05 ^a^	1.03 ± 0.00 ^b^	36.03 ± 0.15 ^fgh^	46.23 ± 0.02 ^cdefghi^	14.73 ± 0.05 ^ab^
	TBHQ/0.1	1.23 ± 0.00 ^c^	23.23 ± 0.10 ^def^	1.64 ± 0.02 ^d^	11.47 ± 0.05 ^fg^	44.53 ± 0.06 ^bcde^	13.71 ± 0.04 ^a^	1.02 ± 0.01 ^b^	35.93 ± 0.15 ^h^	46.17 ± 0.08 ^fghi^	14.73 ± 0.05 ^ab^
	TBHQ/0.2	1.23 ± 0.00 ^c^	23.40 ± 0.07 ^de^	1.60 ± 0.02 ^d^	11.87 ± 0.04 ^ab^	44.38 ± 0.01 ^cdefg^	13.60 ± 0.07 ^ab^	1.02 ± 0.01 ^b^	36.50 ± 0.12 ^bcd^	45.98 ± 0.01 ^hi^	14.62 ± 0.06 ^ab^
	BHT/0.1	1.23 ± 0.00 ^c^	23.42 ± 0.06 ^cd^	1.61 ± 0.04 ^d^	11.89 ± 0.02 ^ab^	44.42 ± 0.03 ^cdef^	13.60 ± 0.06 ^ab^	1.03 ± 0.01 ^b^	36.54 ± 0.08 ^bc^	46.03 ± 0.07 ^ghi^	14.64 ± 0.07 ^ab^
	BHT/0.2	1.24 ± 0.02 ^c^	23.20 ± 0.02 ^def^	1.68 ± 0.02 ^d^	11.42 ± 0.06 ^g^	44.68 ± 0.04 ^abc^	13.64 ± 0.02 ^ab^	1.03 ± 0.01 ^b^	35.86 ± 0.05 ^hi^	46.36 ± 0.06 ^bcdef^	14.67 ± 0.04 ^ab^
	V_E_/0.1	1.26 ± 0.00 ^bc^	23.81 ± 0.08 ^ab^	1.65 ± 0.05 ^d^	11.42 ± 0.04 ^g^	44.88 ± 0.04 ^a^	13.58 ± 0.04 ^ab^	1.02 ± 0.00 ^b^	36.49 ± 0.11 ^bcd^	46.53 ± 0.08 ^abcde^	14.59 ± 0.04 ^ab^
	V_E_/0.2	1.25 ± 0.01 ^bc^	23.98 ± 0.09 ^a^	1.68 ± 0.08 ^d^	11.71 ± 0.03 ^cde^	44.86 ± 0.02 ^a^	13.55 ± 0.01 ^ab^	1.02 ± 0.00 ^b^	36.94 ± 0.07 ^a^	46.55 ± 0.10 ^abc^	14.57 ± 0.01 ^ab^

Note: Values in the same column with different superscript letters differ significantly (*p* < 0.05). SFA, C14:0 + C16:0 + C18:0; MUFA, C16:1 + C18:1; PUFA, C18:2 + C18:3.

**Table 2:** Effects of different antioxidants (g/kg) on the free radical scavenging ability of lard.

	FRAP	ABTS	DPPH-P	DPPH-U	OSI	PI
Control	23.04 ± 1.39 ^c^	115.20 ± 3.82 ^e^	7.50 ± 0.08 ^c^	47.94 ± 2.94 ^f^	0.81 ± 0.01 ^e^	1.00
PG/0.1	170.48 ± 3.77 ^a^	289.11 ± 1.55 ^a^	110.20 ± 3.25 ^a^	148.66 ± 5.05 ^a^	6.27 ± 0.02 ^b^	7.74
TBHQ/0.1	145.29 ± 4.42 ^b^	244.66 ± 2.91 ^b^	94.45 + 5.52 ^b^	110.03 ± 0.66 ^b^	4.11 ± 0.49 ^c^	5.07
TBHQ/0.2	179.43 ± 7.56 ^a^	301.69 ± 2.29 ^a^	109.40 ± 2.12 ^a^	155.68 ± 0.31 ^a^	8.49 ± 0.04 ^a^	10.48
BHT/0.1	28.23 ± 0.27 ^c^	192.72 ± 17.66 ^c^	7.95 ± 0.02 ^c^	80.07 ± 9.65 ^cd^	2.00 ± 0.10 ^d^	2.47
BHT/0.2	33.91 ± 0.11 ^c^	298.35 ± 0.78 ^a^	10.65 ± 0.11 ^c^	83.08 ± 0.16 ^c^	2.55 ± 0.14 ^d^	3.15
VE/0.1	26.20 ± 0.32 ^c^	133.91 ± 6.16 ^d^	6.93 ± 0.53 ^c^	63.49 ± 0.85 ^e^	0.90 ± 0.07 ^e^	1.11
VE/0.2	33.86 ± 1.62 ^c^	154.29 ± 8.53 ^d^	7.92 ± 0.44 ^c^	68.37 ± 1.35 ^de^	0.83 ± 0.07 ^e^	1.02

Note: All measurements were performed after 15 days of accelerated oxidation at 60 °C. Values in the same column with different superscript letters differ significantly (*p* < 0.05).

**Table 3:** Effects of different antioxidants (g/kg) on tocopherols during accelerated oxidation of lard.

Day	Antioxidants	α-Tocopherol	β-Tocopherol	γ-Tocopherol	δ-Tocopherol	Total Tocopherols
0 d	VE/0.1	41.14 ± 0.13 ^b^	10.65 ± 0.00 ^b^	10.98 ± 0.04 ^b^	27.64 ± 1.98 ^b^	90.41 ± 1.89 ^b^
	VE/0.2	85.38 ± 0.20 ^a^	14.97 ± 0.15 ^a^	20.93 ± 0.56 ^a^	54.91 ± 0.16 ^a^	176.19 ± 0.35 ^a^
3 d	VE/0.1	9.71 ± 0.17 ^d^	N. D.	N. D.	N. D.	9.71 ± 0.17 ^cd^
	VE/0.2	10.85 ± 0.11 ^c^	N. D.	N. D.	N. D.	10.85 ± 0.11 ^c^
6 d	VE/0.1	8.22 ± 0.03 ^e^	N. D.	N. D.	N. D.	8.22 ± 0.03 ^de^
	VE/0.2	7.69 ± 0.45 ^e^	N. D.	N. D.	N. D.	7.69 ± 0.45 ^e^
9 d	VE/0.1	5.53 ± 0.06 ^f^	N. D.	N. D.	N. D.	5.53 ± 0.06 ^f^
	VE/0.2	4.17 ± 0.11 ^g^	N. D.	N. D.	N. D.	4.17 ± 0.11 ^fg^
12 d	VE/0.1	4.51 ± 0.09 ^g^	N. D.	N. D.	N. D.	4.51 ± 0.09 ^fg^
	VE/0.2	4.17 ± 0.04 ^g^	N. D.	N. D.	N. D.	4.17 ± 0.04 ^fg^
15 d	VE/0.1	3.93 ± 0.07 ^g^	N. D.	N. D.	N. D.	3.93 ± 0.07 ^fg^
	VE/0.2	3.29 ± 0.25 ^h^	N. D.	N. D.	N. D.	3.29 ± 0.25 ^g^

Note: Values in the same column with different superscript letters differ significantly (*p* < 0.05).

**Table 4:** Ranking table of principal component analysis scores of different antioxidants (g/kg) in lard.

Antioxidants	F1 Score	F1 Ranking	F2 Score	F2 Ranking	Fn Score	Fn Ranking
Control	−3.039	8	−0.506	5	−2.684	8
PG/0.1	3.047	2	−0.537	6	2.545	2
TBHQ/0.1	1.888	3	0.101	3	1.638	3
TBHQ/0.2	4.449	1	−0.463	4	3.761	1
BHT/0.1	−1.023	5	2.156	1	−0.577	5
BHT/0.2	−0.505	4	1.237	2	−0.261	4
VE/0.1	−2.470	7	−1.043	8	−2.270	7
VE/0.2	−2.348	6	−0.945	7	−2.152	6

## Data Availability

The original contributions presented in this study are included in the article. Further inquiries can be directed to the corresponding author.

## Abbreviations

TBHQ	Tert-butylhydroquinone
PG	Propyl gallate
BHT	Butylated hydroxytoluene
VE	Vitamin E
EPR	Electron Paramagnetic Resonance
AV	Acid value
POV	Peroxide value
P-AnV	P-anisidine value
